# Exceptional laceration of flexor digitorum tendons proximal to a severe palmar hand wound: a case report with literature review

**DOI:** 10.11604/pamj.2015.22.266.7495

**Published:** 2015-11-20

**Authors:** Badr Ennaciri, Mustapha Mahfoud, Ahmed El Bardouni, Mohamed Saleh Berrada

**Affiliations:** 1 Department of Orthopedics, Avicenna University Hospital, Rabat, Morocco

**Keywords:** Flexor tendons, Kessler suture, hand wound

## Abstract

Hand wounds are common, poor functional outcomes are marked because of sequelae inherent to posttraumatic and postoperative complications. Suitable surgery repair in emergency can ensure best results. Classically, tendon's injuries occur near the injured area and their repair depend on traumatized zone, sutures techniques, associated lesions and surgeon's abilities. We report a case of a farmer who has sustained of a severe hand wound due to blades of a combine harvester. Clinical examination showed exceptional laceration of 2^nd^ and 3^rd^ flexor digitorum tendons from musculo-tendinous junction, without any lesion in their palmar section. We proceeded; after extensive debridement, abundant lavage and removal of foreign body; to modified Kessler sutures using PDS 4.0 followed by dorsal splint for protecting tendons repair, and progressive rehabilitation program. Final result was interesting after 12 weeks. Thinking to tendon laceration is important, when manipulating machines with rotational movements.

## Introduction

Hand wounds represent 10-15% of admissions in emergency departments in developed countries [1]. Complex lesions require urgent treatment after good clinical and radiological exams. The objective of this assessment is to adopt correct guideline for preventing functional and aesthetic sequelae. Avoiding such severe traumatisms need prevention in workplaces [2]. Surgical repair of sectioned flexor tendons has been improved in the last 40 years thanks to a better knowledge of anatomy, physiology, the development of new suture techniques and specific rehabilitation program. We report the case of a farmer admitted to emergency department at Avicenna university hospital in Rabat, Morocco, for circumferential deep wound of the right hand with exceptional laceration of the 2^nd^ and 3^rd^ flexor digitorum tendons from the palmar zone V.

## Patient and observation

**Figure 1:**
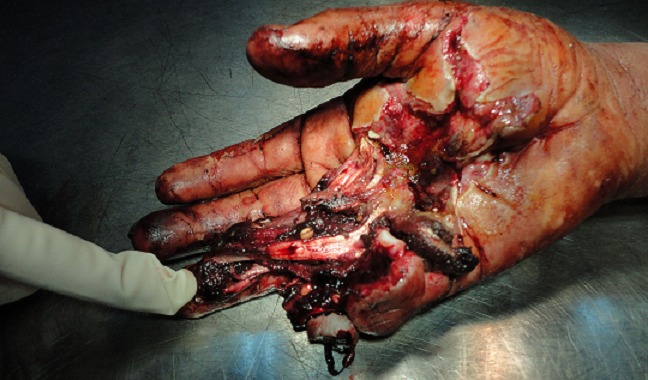
Deep, defiled and circumferential wound interesting Zone III and the ulnar border of the right hand associated to an open distal interphalangeal joint dislocation of the 5th finger

**Figure 2:**
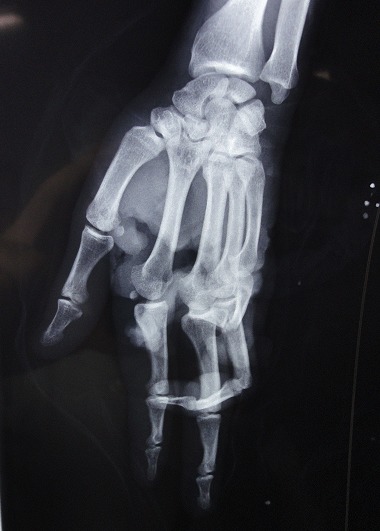
Radiograph of the right hand showing distal interphalangeal joint dislocation of the 5th finger

**Figure 3:**
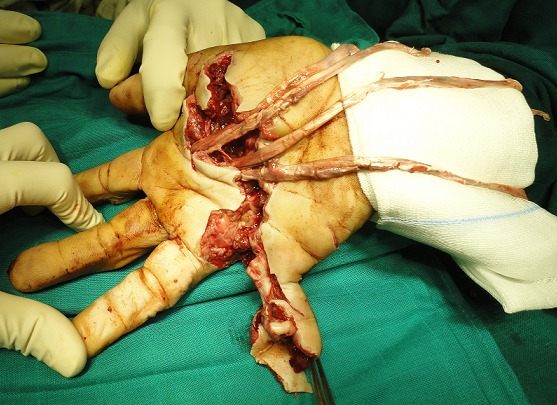
Surgical view of the right hand showing laceration of the 2nd and 3rd flexor tendons, after extensive debridement of non-viable tissue, abundant lavage and removal of foreign body

**Figure 4:**
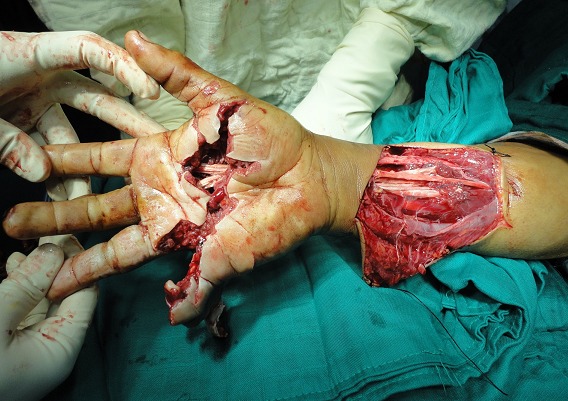
Flexor tendons repair with modified Kessler sutures in musculo-tendinous junction

**Figure 5:**
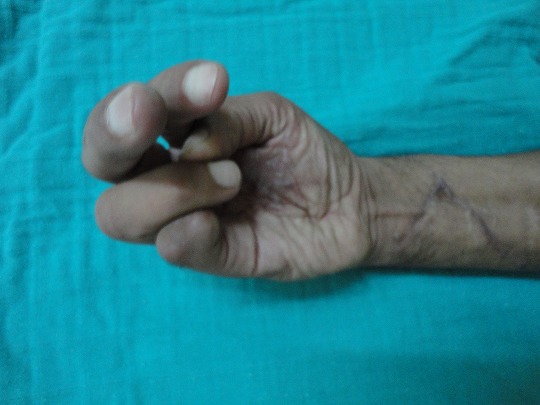
Restoration of finger flexion

**Figure 6:**
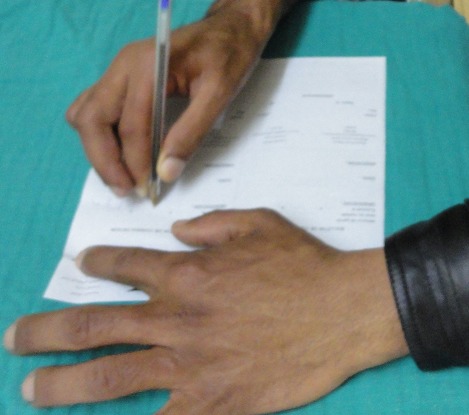
Restoration of functional right hand

A 38 years old man, farmer, right-handed. He has sustained of a deep wound of the right hand after a work accident by the blades of a combine harvester. General exam at admission in emergency department showed stable hemodynamic status; right hand examination objectified deep, defiled, and circumferential wound interesting Zone III and the ulnar border, there was no fractures but open distal interphalangeal joint dislocation of the 5^th^ finger; flexor tendons were intact in this zone, but abnormally wrapped; flexion of the 2^nd^ and 3^rd^ fingers was impossible; neuro-vascular exam showed digital hypoesthesia without pulp ischemia (Figure 1). Radiograph of the right hand showed distal interphalangeal joint dislocation of the 5^th^ finger (Figure 2). After tetanus serum injection, the patient was transferred to the operating room, where hemostasis was addressed with electrocautery, concurrently with extensive debridement of non-viable tissue and abundant lavage with removal of foreign body (Figure 3). Both, 2^nd^ and 3^rd^ *flexor digitorum superficialis and profundus* tendons were lacerated from musculo-tendinous junction. We realized anterior approach of the distal forearm, followed by modified Kessler sutures using PDS 4.0 of the sectioned tendons (Figure 4). Curative antibiotics, local care were prescribed to its output. A dorsal hand and wrist protective splint was applied during 6 weeks, metacarpophalangeal and interphalangeal joints in flexion. On postoperative day, passive and progressive exercises for the fingers were performed; the rehabilitation process consisted of active extension and flexion during 6 weeks. After 3 month, follow-up showed interesting results; apart from keloid palmar scar and 5^th^ finger retraction, functional hand was restored without any infection stigmata (Figure 5, Figure 6).

## Discussion

The wounds of the hand constitute a real surgical emergency. They are commonly seen in young working class and related to work injuries and to domestic accidents [3]. The socioeconomic impact is important. Dexterity and complexity of this organ make any surgery repair more difficult and sequelae are often obvious. All vascular, nervous, tendinous and osseous structures can be injured depending on wounding strength and agent. Objective examination appreciates the age of the patient, causes of the injury, wounding agent, time since injury; the dimensions of the wound, tendons status, vascular and neurological status. Radiographs of the hand are fundamental to diagnosis fractures, dislocations, tendons and ligament avulsions. Flexor digitorum tendons are usually damaged and consequently can deteriorate the hand function. Most wounds of flexor tendons occur in flected finger and tendon section is located near the injured area [4]. Obtaining best outcome need primary surgical repair in emergency with restoration of length, strength and gliding excursion of tendon followed by post-operative rehabilitation. Palmar area is divided into V zones. Zone V extends from the proximal border of the transverse carpal ligament to the musculotendinous junction in the proximal part of the forearm; tendon laceration in this area made its repair easy by a modified dual Kessler using PDS 4.0 in our case. Modified Kessler technique reinforced with continues running suture can provide enough strength at flexor tendon repair site to permit early gentle passive and active motion of the fingers [5]. Commonly, the suture techniques are composed of core sutures and peripheral sutures. A new modified Tsuge technique seem to be interesting for flexor tendon repair [6]. Using non absorbable sutures or absorbable ones are controversial. The use of absorbable sutures limits foreign body implantation such as excessive fibrosis and granuloma, but maintaining adequate tensile strength is unpredictable due to their absorption into the body [7]. Non absorbable sutures present advantages of easy handling, good biocompatibility, and minimal loss of tensile strength after knotting. O'Broin [8] demonstrated that PDS was flexible, strong, had high breaking strength (9 weeks). Postoperative complications such as infection, adhesion and joint stiffness may occur after flexor tendon repair. Early active mobilization permit restoration of tendon course [9]. In our case report, elevation minimized swelling and pain; antibiotics prevented infection; analgesia controlled pain and physiotherapy helped early return to full function.

## Conclusion

Hand injuries are common and lead to heavy financial loads in terms of treatment, job loss, and time of duty.Targeted campaigns to sensitize workplaces may contribute to prevention of hand injuries. Tendon laceration, in a wounded hand, is rare and must be considered when patients manipulate machines with rotational movements.

## References

[1] Vadivelu R, Dias JJ, Burke FD, Stanton J (2006). Handinjuries in children: a prospective study. J PediatrOrthop..

[2] Tan KK, Fishwick NG, Dickson WA, Sykes PJ (1991). Does training reduce the incidence of industrial hand injuries?. J Hand Surg Br..

[3] Dubert T, Allieu Y, Bellemère P, Egloff D, Nonnenmacher J, Baudet J (2003). Huit jours d'urgences mains: rapport de l'auditréalisé dans les centres FESUM du 3 au 9 juin 2002. Chir Main..

[4] Le Nen D, Hu W, Dartoy C, Guyot X, Lefèvre C (1999). Plaies de la main: EMC Apparei llocomoteur.

[5] Nasab Seyed Abdolhossein Mehdi, Sarrafan Nasser, Saeidian Seyed Reza, Emami Hassan (2013). Functional outcome of flexor tendon repair of the hand at Zone 5 and post-operativeearly mobilization of the fingers. Pak J Med Sci..

[6] Jianghai Chen, Kun Wang, Foad Katirai, Zhenbing Chen (2014). A new modified Tsuge suture for flexor tendon repairs: the biomechanical analysis and clinical application. J Orthop Surg Res..

[7] Wada A, Kubota H, Akiyama T, Hatanaka H, Miura H, Iwamoto Y (2001). Effect of absorbable polydioxanone flexor tendon repair and restricted active mobilization in a canine model. J Hand Surg Am..

[8] O'Broin ES, Earley MJ, Smyth H, Hooper AC (1995). Absorbable sutures in tendon repair: a comparison of PDS with prolene in rabbit tendon repair. J Hand Surg Br..

[9] Starr HM, Snoddy M, Hammond KE, Seiler JG (2013). Flexor tendon repair rehabilitation protocols: a systematic review. J Hand Surg Am..

